# Supplementary material for: Genomic Analysis of Vavilov’s Historic Chickpea Landraces Reveals Footprints of Environmental and Human Selection
Source: Int J Mol Sci. 2020 May 31;21(11):3952. doi: 10.3390/ijms21113952 (PMC7313079; doi:10.3390/ijms21113952)
Supplement: Supplementary file 1 [file ijms-21-03952-s001.zip › Supplementary Figures S1-S20.pdf]

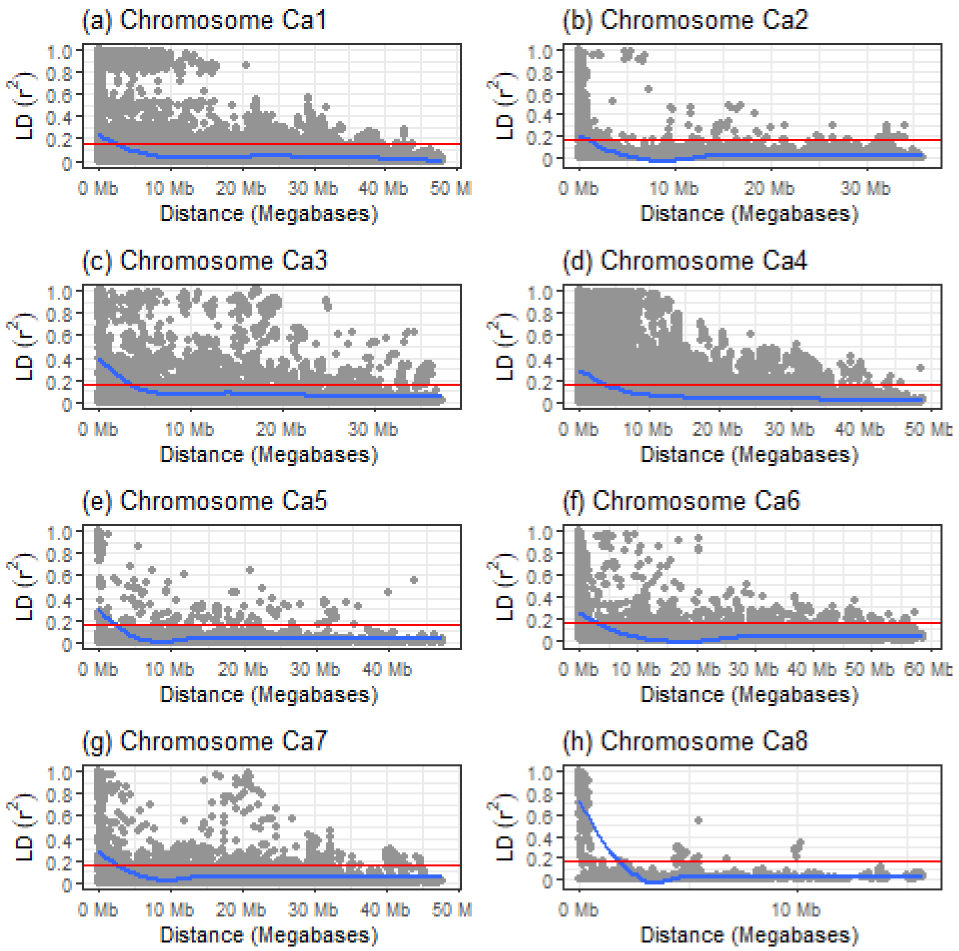


**Figure S1. Linkage disequilibrium ($r^2$) plots for the each chromosome of chickpea genome. The horizontal red line indicates the 95th percentile of the distribution of the unlinked $r^2$, which gives the critical value of $r^2$.**

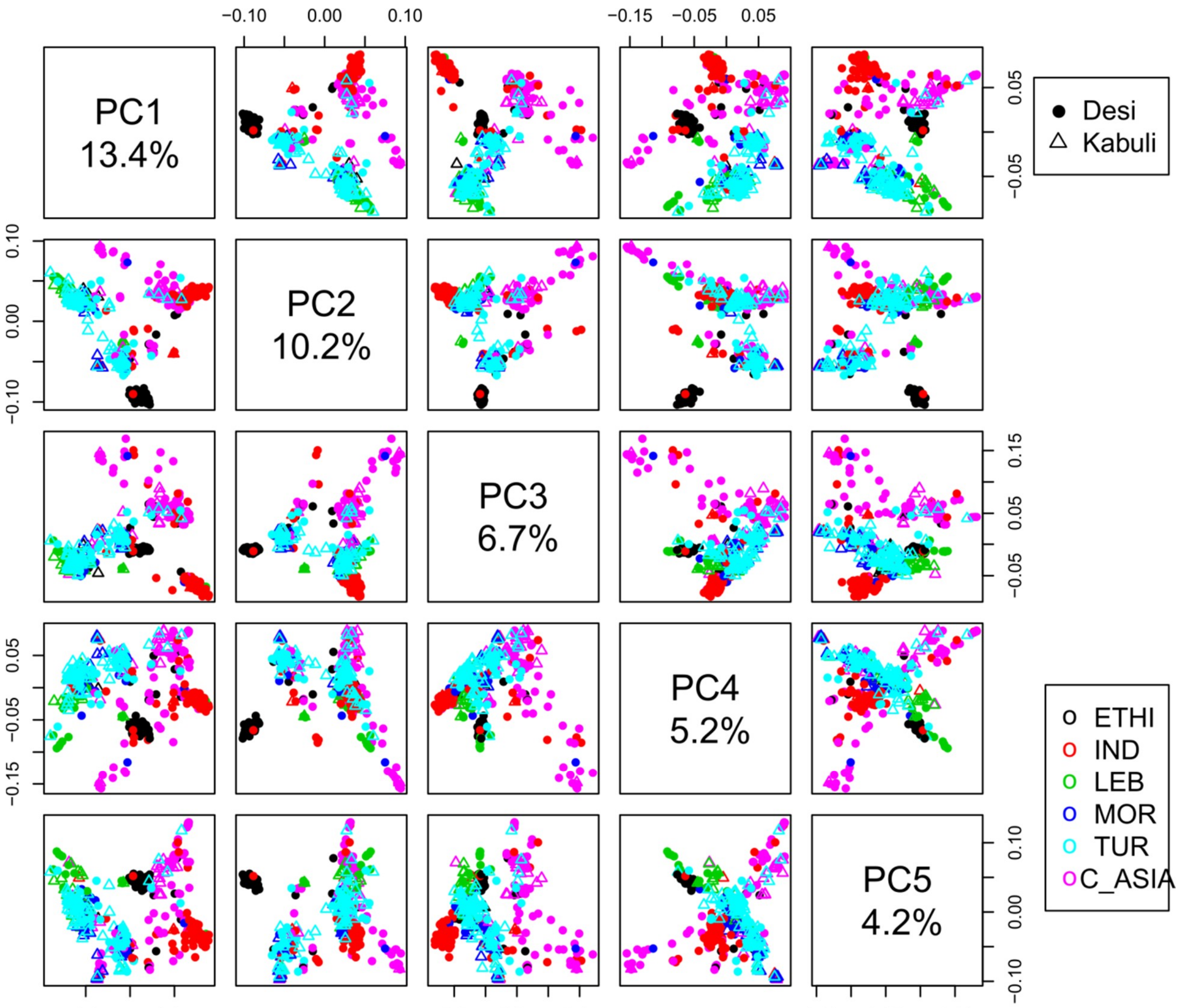


**Figure S2. Scatter plots of the first five principal components of PCA analysis based on 2,579 SNPs. Each dot represents an accession. Desi varieties are shown as circles and Kabuli as triangles.**

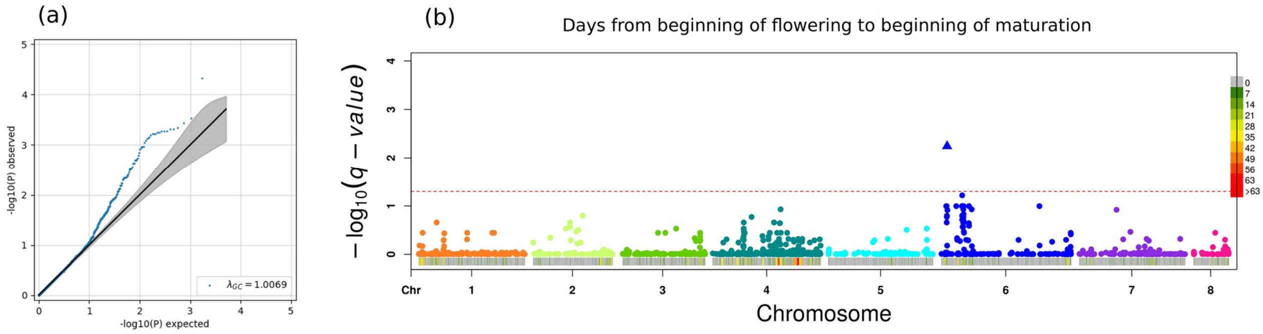


**Figure S3. Summary of GWAS analyses with eight PCs as covariates for phenotypic trait Days from beginning of flowering to beginning of maturation.** (a) SNP QQ-plot. (b) SNP Manhattan plot (different colors correspond to different chromosomes). SNPs with q-value <

0.05 are shown for each chromosome, marked as triangles. Chromosome density is attached on the bottom of Manhattan plot.

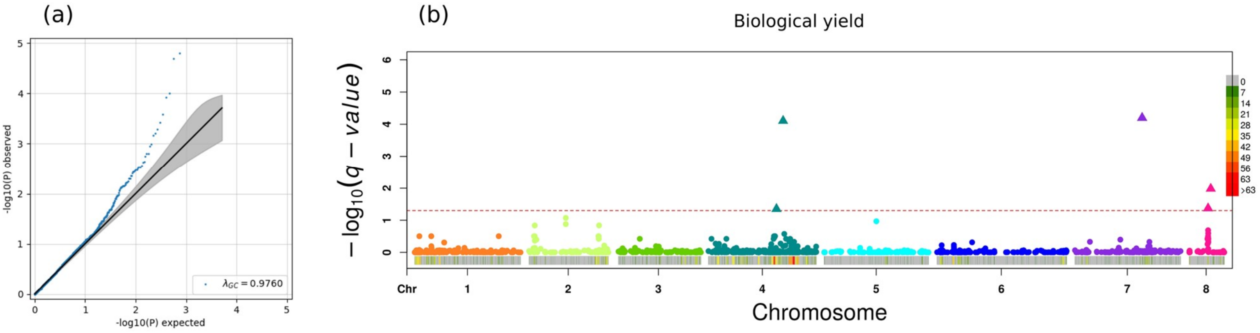


**Figure S4. Summary of GWAS analyses with eight PCs as covariates for phenotypic trait Biological yield.** (a) SNP QQ-plot. (b) SNP Manhattan plot (different colors correspond to different chromosomes). SNPs with q-value < 0.05 are shown for each chromosome, marked as triangles. Chromosome density is attached on the bottom of Manhattan plot.

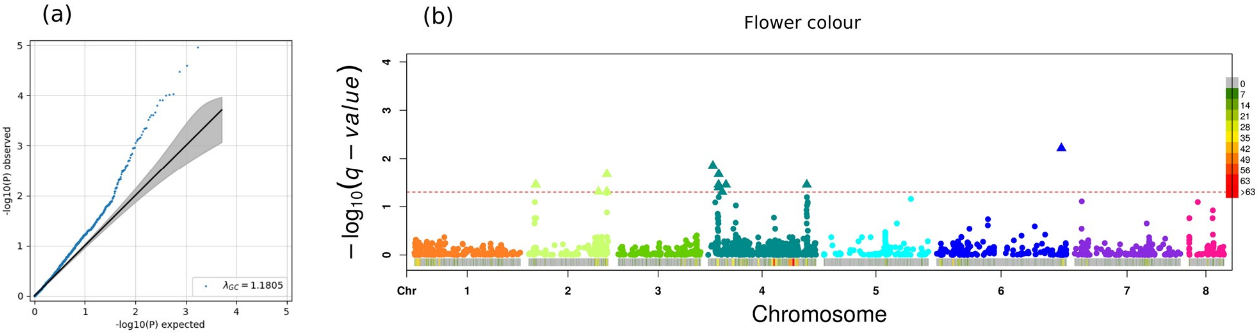


**Figure S5. Summary of GWAS analyses with eight PCs as covariates for phenotypic trait Flower colour.** (a) SNP QQ-plot. (b) SNP Manhattan plot (different colors correspond to different chromosomes). SNPs with q-value < 0.05 are shown for each chromosome, marked as triangles. Chromosome density is attached on the bottom of Manhattan plot.

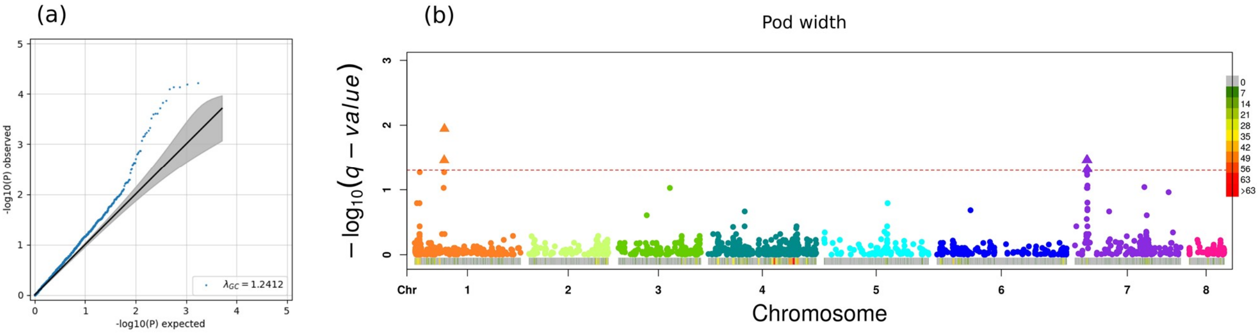

**Figure S6. Summary of GWAS analyses with eight PCs as covariates for phenotypic trait Pod width.** (a) SNP QQ-plot. (b) SNP Manhattan plot (different colors correspond to different chromosomes). SNPs with q-value < 0.05 are shown for each chromosome, marked as triangles. Chromosome density is attached on the bottom of Manhattan plot.

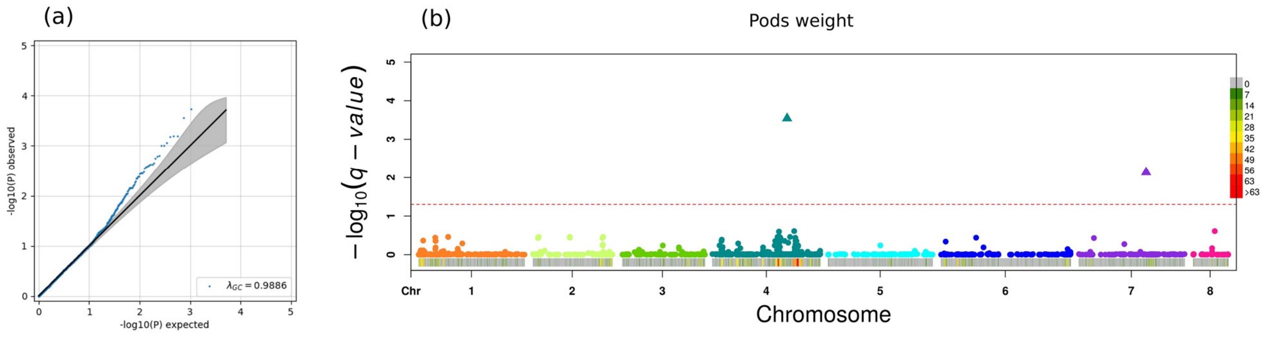


**Figure S7. Summary of GWAS analyses with eight PCs as covariates for phenotypic trait Pods weight.** (a) SNP QQ-plot. (b) SNP Manhattan plot (different colors correspond to different chromosomes). SNPs with q-value < 0.05 are shown for each chromosome, marked as triangles. Chromosome density is attached on the bottom of Manhattan plot.

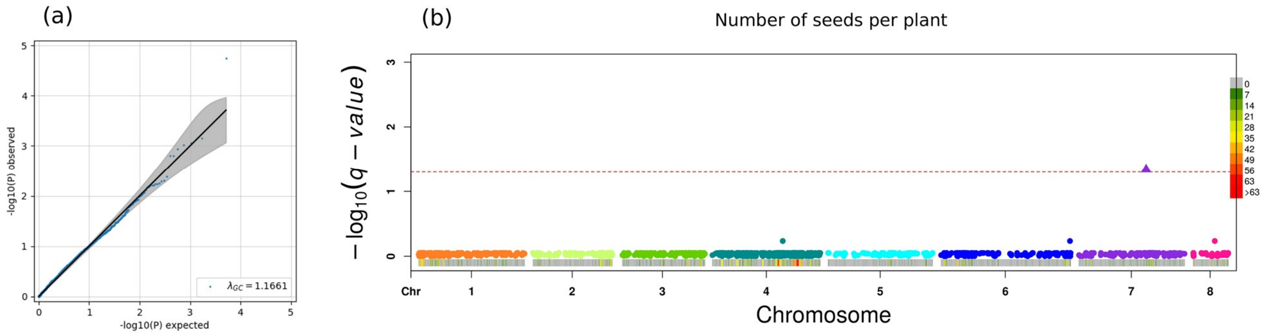


**Figure S8. Summary of GWAS analyses with eight PCs as covariates for phenotypic trait Number of seeds per plant.** (a) SNP QQ-plot. (b) SNP Manhattan plot (different colors correspond to different chromosomes). SNPs with q-value < 0.05 are shown for each chromosome, marked as triangles. Chromosome density is attached on the bottom of Manhattan plot.

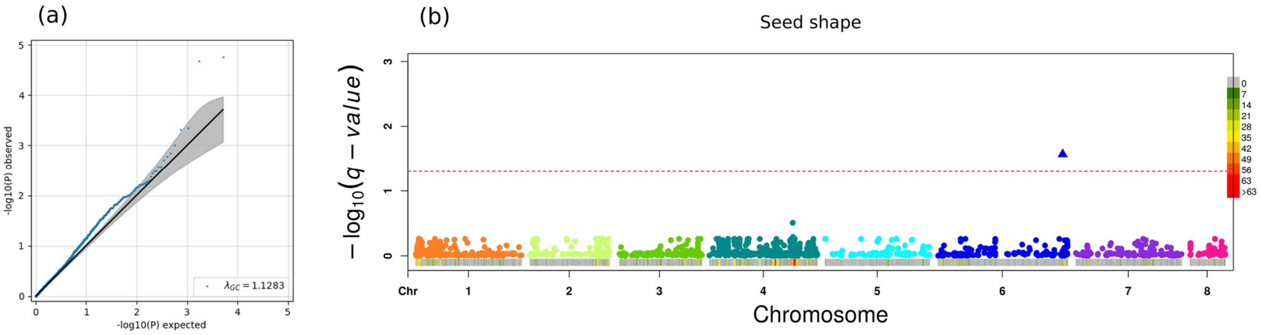


**Figure S9. Summary of GWAS analyses with eight PCs as covariates for phenotypic trait Seed shape.** (a) SNP QQ-plot. (b) SNP Manhattan plot (different colors correspond to different chromosomes). SNPs with q-value < 0.05 are shown for each chromosome, marked as triangles. Chromosome density is attached on the bottom of Manhattan plot.

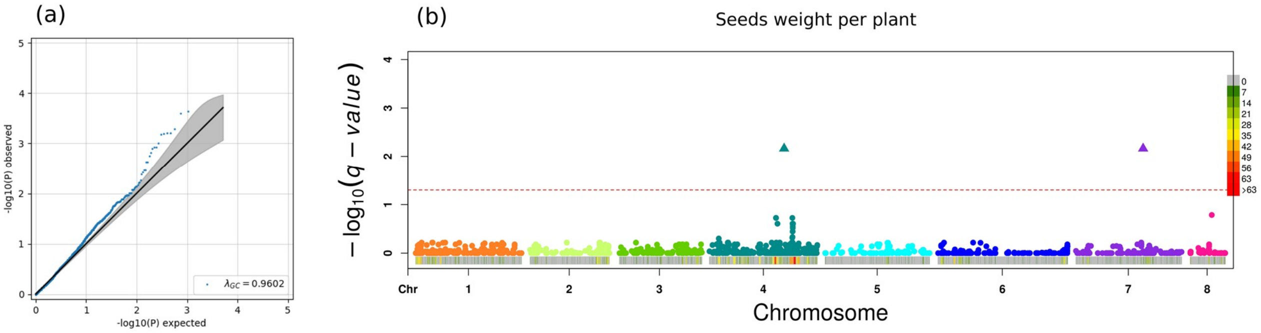


**Figure S10. Summary of GWAS analyses with eight PCs as covariates for phenotypic trait Seeds weight per plant.** (a) SNP QQ-plot. (b) SNP Manhattan plot (different colors correspond to different chromosomes). SNPs with q-value < 0.05 are shown for each chromosome, marked as triangles. Chromosome density is attached on the bottom of Manhattan plot.

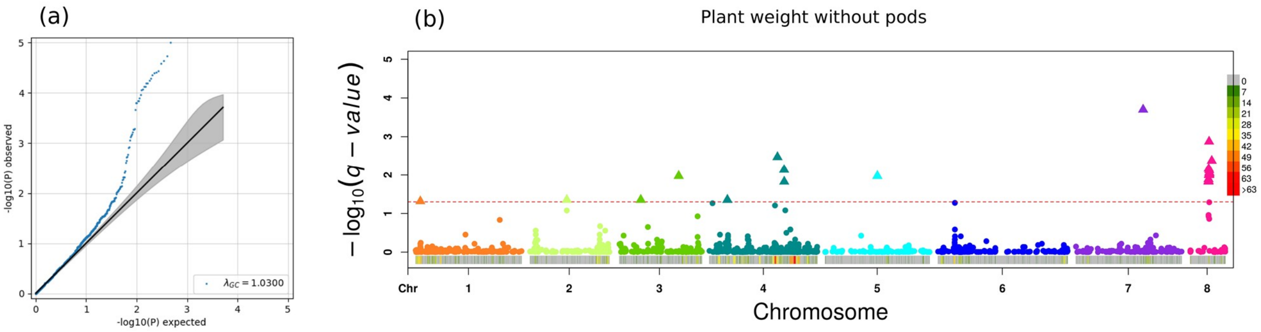


**Figure S11. Summary of GWAS analyses with eight PCs as covariates for phenotypic trait Plant weight without pods.** (a) SNP QQ-plot. (b) SNP Manhattan plot (different colors

correspond to different chromosomes). SNPs with q-value < 0.05 are shown for each chromosome, marked as triangles. Chromosome density is attached on the bottom of Manhattan plot.

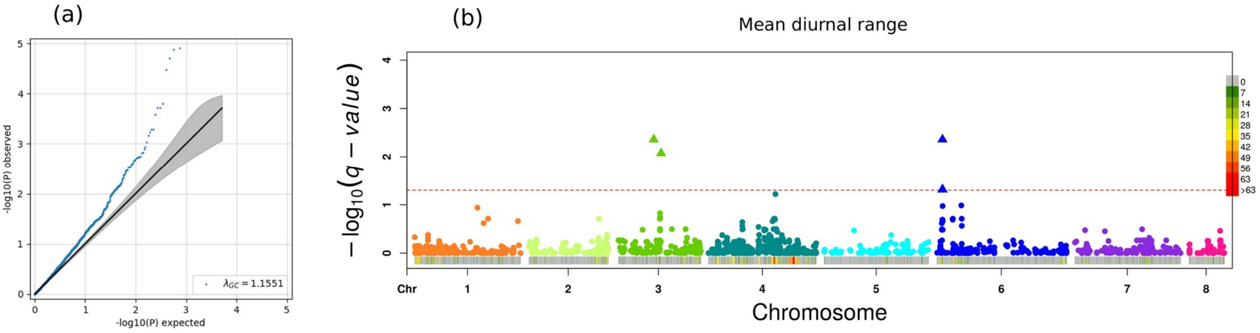


**Figure S12. Summary of GWAS analyses with eight PCs as covariates for bioclimatic variable Mean diurnal range.** (a) SNP QQ-plot. (b) SNP Manhattan plot (different colors correspond to different chromosomes). SNPs with q-value < 0.05 are shown for each chromosome, marked as triangles. Chromosome density is attached on the bottom of Manhattan plot.

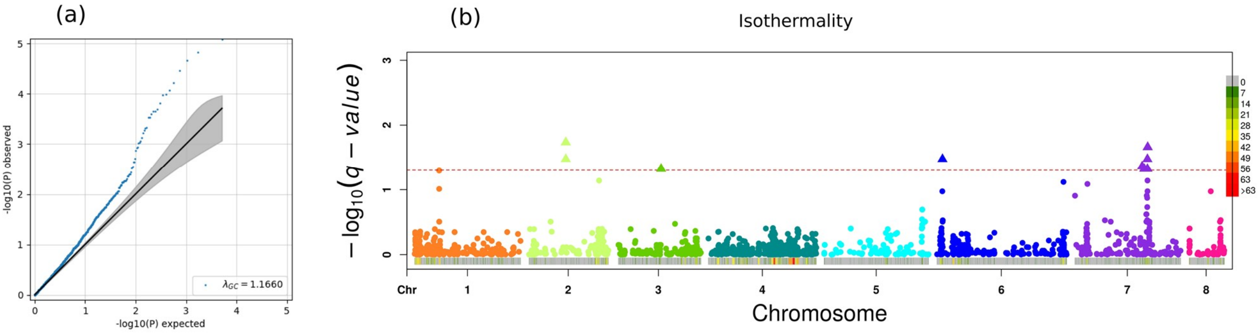


**Figure S13. Summary of GWAS analyses with eight PCs as covariates for bioclimatic variable Isothermality.** (a) SNP QQ-plot. (b) SNP Manhattan plot (different colors correspond to different chromosomes). SNPs with q-value < 0.05 are shown for each chromosome, marked as triangles. Chromosome density is attached on the bottom of Manhattan plot.

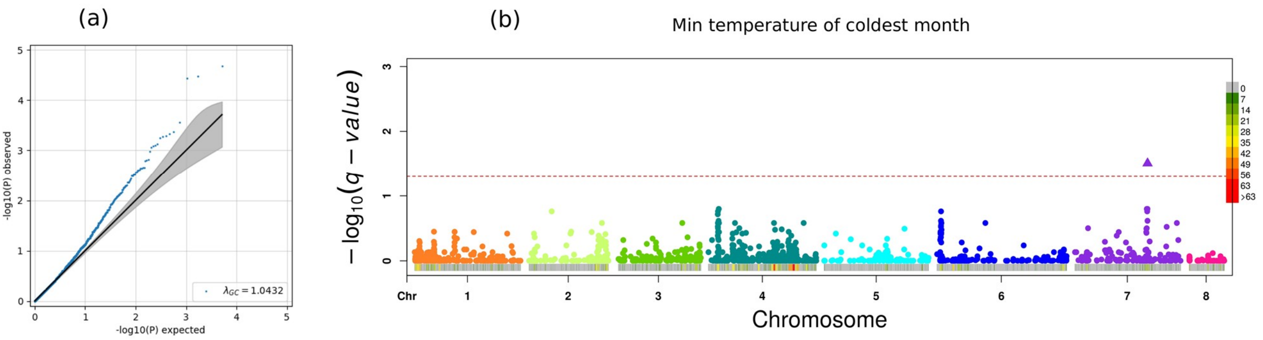


**Figure S14. Summary of GWAS analyses with eight PCs as covariates for bioclimatic variable Min temperature of coldest month.** (a) SNP QQ-plot. (b) SNP Manhattan plot (different colors correspond to different chromosomes). SNPs with q-value < 0.05 are shown for each chromosome, marked as triangles. Chromosome density is attached on the bottom of Manhattan plot.

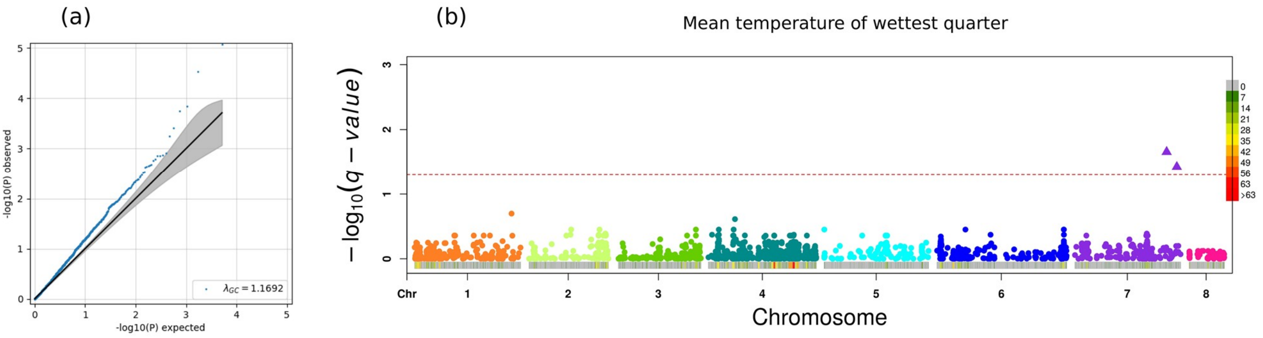


**Figure S15. Summary of GWAS analyses with 8 PCs as covariates for bioclimatic variable Mean temperature of wettest quarter.** (a) SNP QQ-plot. (b) SNP Manhattan plot (different colors correspond to different chromosomes). SNPs with q-value < 0.05 are shown for each chromosome, marked as triangles. Chromosome density is attached on the bottom of Manhattan plot.

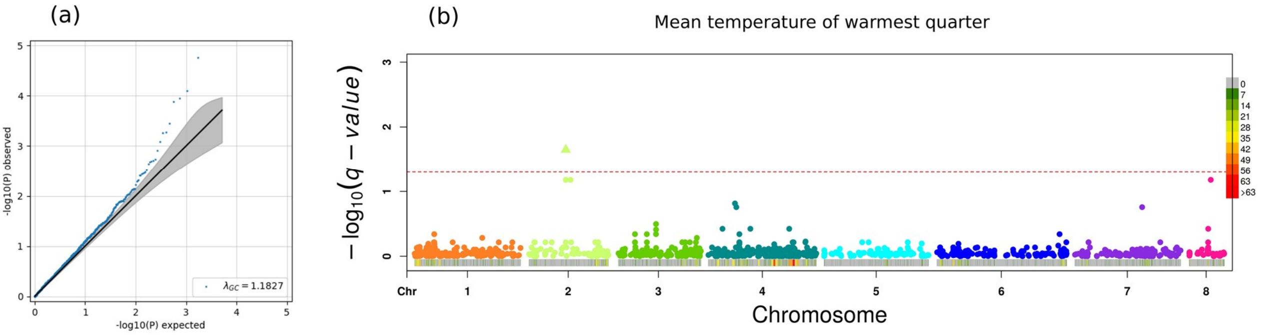

**Figure S16. Summary of GWAS analyses with 8 PCs as covariates for bioclimatic variable Mean temperature of warmest quarter.** (a) SNP QQ-plot. (b) SNP Manhattan plot (different colors correspond to different chromosomes). SNPs with q-value < 0.05 are shown for each chromosome, marked as triangles. Chromosome density is attached on the bottom of Manhattan plot.

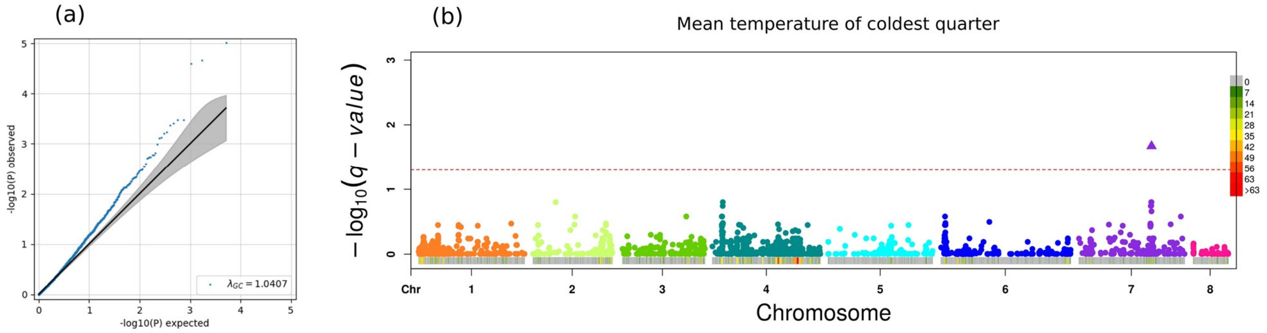


**Figure S17. Summary of GWAS analyses with 8 PCs as covariates for bioclimatic variable Mean temperature of coldest quarter.** (a) SNP QQ-plot. (b) SNP Manhattan plot (different colors correspond to different chromosomes). SNPs with q-value < 0.05 are shown for each chromosome, marked as triangles. Chromosome density is attached on the bottom of Manhattan plot.

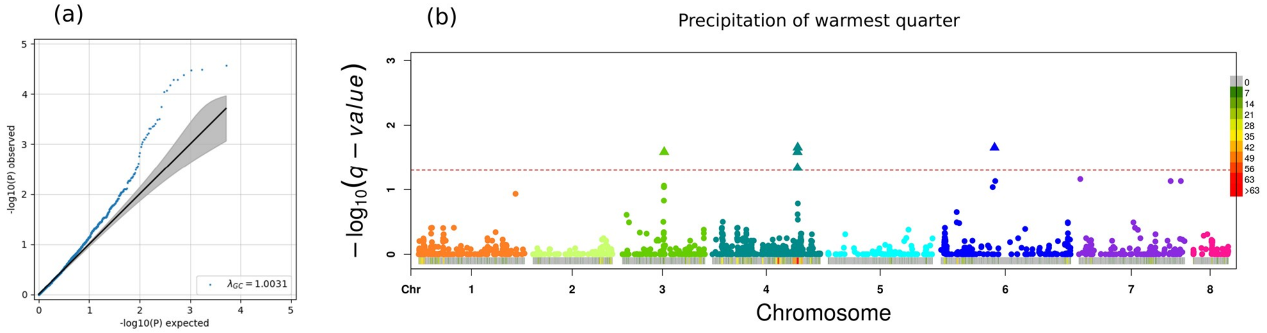


**Figure S18. Summary of GWAS analyses with eight PCs as covariates for bioclimatic variable Precipitation of warmest quarter.** (a) SNP QQ-plot. (b) SNP Manhattan plot (different colors correspond to different chromosomes). SNPs with q-value < 0.05 are shown for

each chromosome, marked as triangles. Chromosome density is attached on the bottom of Manhattan plot.

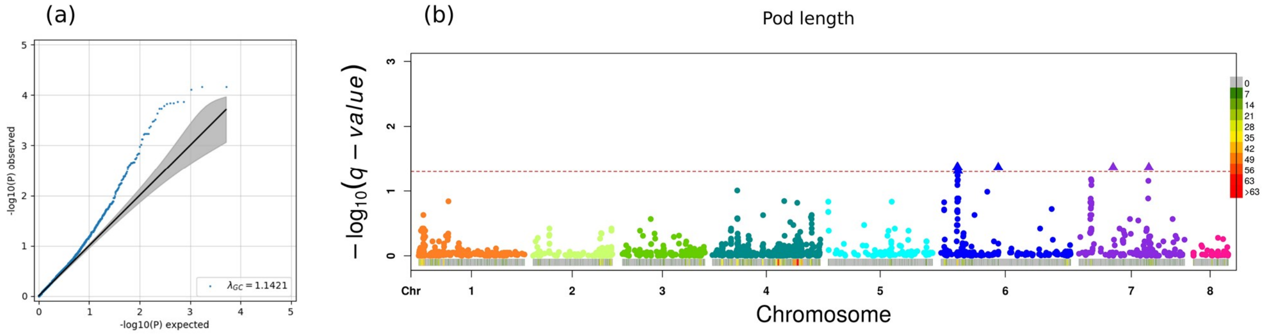


**Figure S19. Summary of GWAS analyses with eight PCs and 2 PCoAs as covariates for phenotypic trait Pod length.** (a) SNP QQ-plot. (b) SNP Manhattan plot (different colors correspond to different chromosomes). SNPs with q-value < 0.05 are shown for each chromosome, marked as triangles. Chromosome density is attached on the bottom of Manhattan plot.

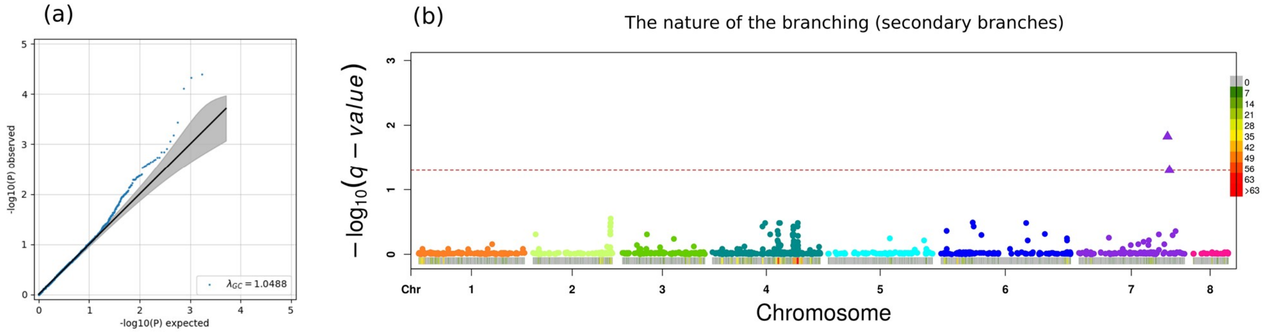


**Figure S20. Summary of GWAS analyses with eight PCs and 2 PCoAs as covariates for phenotypic trait of branching (secondary branches).** (a) SNP QQ-plot. (b) SNP Manhattan plot (different colors correspond to different chromosomes). SNPs with q-value < 0.05 are shown for each chromosome, marked as triangles. Chromosome density is attached on the bottom of Manhattan plot.
