# Supplementary material for: Genomic Analysis of Vavilov’s Historic Chickpea Landraces Reveals Footprints of Environmental and Human Selection
Source: Int J Mol Sci. 2020 May 31;21(11):3952. doi: 10.3390/ijms21113952 (PMC7313079; doi:10.3390/ijms21113952)
Supplement: Supplementary file 1 [file ijms-21-03952-s001.zip › Supplementary Tables S1-S12.pdf]

Table S1 List of accessions, collection sites and accession's groups.

(see TableS1.xlsx)

Table S2 List of bioclimatic variables and their abbreviations.

**Bioclimatic variable**	**Abbreviation**
Annual mean temperature, °C * 10	BIO_1
Mean diurnal range, °C * 10	BIO_2
Isothermality, %	BIO_3
Temperature seasonality, standard deviation *100	BIO_4
Max temperature of warmest month, °C * 10	BIO_5
Min temperature of coldest month, °C * 10	BIO_6
Temperature annual range, °C * 10	BIO_7
Mean temperature of wettest quarter, °C * 10	BIO_8
Mean temperature of warmest quarter, °C * 10	BIO_{10}
Mean temperature of coldest quarter, °C * 10	BIO_{11}
Annual precipitation, mm	BIO_{12}
Precipitation of wettest month, mm	BIO_{13}
Precipitation of driest month, mm	BIO_{14}
Precipitation seasonality, mm	BIO_{15}
Precipitation of wettest quarter, mm	BIO_{16}
Precipitation of driest quarter, mm	BIO_{17}

Precipitation of warmest quarter, mm	BIO_{18}
Precipitation of coldest quarter, mm	BIO_{19}
Digital elevation model, m	DEM

Table S3 Groups of correlated bioclimatic variables.

**Group**	**Bioclimatic variables**
Group №1	BIO_1, BIO_3, BIO_6, BIO_8, BIO_{11}, BIO_{15}
Group №2	BIO_{12}, BIO_{13}, BIO_{16}, BIO_{18}
Group №3	BIO_5, BIO_{10}
Group №4	BIO_{14}, BIO_{17}
Group №5	BIO_4, BIO_7

Table S4 List of phenotypes and their abbreviations.

**Phenotype**	**Abbreviation**
Ascochyta resistance, score	AscoRes
Ascochyta the degree of damage, score	AsoDes
Biological yield, g	Byld
Bush shape, score	BushShape
Days from end of flowering to beginning of maturation, days	EndFloBegMatu
Days from beginning of flowering to beginning of maturation, days	BegFloBegMatu
Days from beginning of flowering to end of flowering, days	BegFloEndFlo

Days from beginning of maturation to end of maturation, days	BegMatuEndMatu
Days from beginning of germination to end of germination, days	Germ
Days from beginning of germination to beginning of flowering, days	GermBegFlo
Days from beginning of germination to beginning of maturation, days	GermBegMatu
Days from beginning of germination to end of flowering, days	GermEndFlo
Days from beginning of germination to end of maturation, days	GermEndMatu
Flower colour, score	FloCol
Height of lower pod attachment, cm	Hlp
Leaf size, score	SizeLeaf
Length of the primary branches, score	StemLength1order
Number of pods per plant, PCs	PPP
Number of seeds per plant, PCs	SPP
Peduncle colour, score	FlowstemColo
Plant height, cm	Ptht
Plant weight without pods, g	WpWp
Pods weight, g	PodsWeight
Pod length, mm	PDL
Pod width, mm	PDW
Pod drop, score	PodDrop
Pod shape, score	PodSH
Pod shattering, score	PodShat

Seed colour, score	SCO
Seed shape, score	SSH
Seeds weight per plant, g	SYDS
Stem branchness, score	StemBranchness
Stem colour, score	StemColo
The nature of the branching (secondary branches), score	StemBranch2order
The nature of the branching (primary branches), score	StemBranchiness1order
Thousand seeds weight, g	TSW

Table S5 The nucleotide diversity (pi) estimated separately for each chromosome and geographical group.

Chromosome	Ethiopia	India	Lebanon	Morocco	Turkey	Central Asia
1	0.11*	0.21	0.25	0.23	0.18	0.26
2	0.16	0.26	0.26	0.29	0.30	0.31
3	0.07	0.19	0.19	0.15	0.14	0.29
4	0.16	0.21	0.22	0.29	0.29	0.30
5	0.13	0.19	0.21	0.23	0.19	0.26
6	0.11	0.23	0.15	0.15	0.15	0.26
7	0.15	0.31	0.22	0.21	0.22	0.27
8	0.18	0.22	0.27	0.25	0.29	0.31

* - differences between Ethiopia and other geographical group are statistically significant for each chromosome, the same holds for Central Asian group.

Table S6 The proportion of variance in a phenotype explained by all GWAS SNPs (i.e. the SNP-based heritability) for phenotypic data.

Phenotype	Genotype variation (SE*)	Phenotype variation (SE*)	SNP-based heritability (SE*)	p-value
Days from end of flowering to beginning of maturation	0.004129 (0.001526)	0.027148 (0.001938)	0.152087 (0.053459)	2.978e-09
Pods weight	0.002590 (0.001024)	0.023239 (0.001640)	0.111450 (0.042771)	1.246e-12
Ascochyta resistance	0.012743 (0.004685)	0.096856 (0.006859)	0.131571 (0.046425)	2.837e-14
Ascochyta the degree of damage	0.012743 (0.004685)	0.096856 (0.006859)	0.131571 (0.046425)	2.837e-14
Days from beginning of flowering to beginning of maturation	0.000977 (0.000482)	0.012585 (0.000887)	0.077594 (0.037848)	0.0002352
Days from beginning of	0.002113 (0.000891)	0.017793 (0.001259)	0.118763 (0.048552)	3.774e-07

flowering to end of flowering				
Days from beginning of maturation to end of maturation	0.000674 (0.000304)	0.008940 (0.000630)	0.075381 (0.033571)	2.65e-07
Bush shape	0.007024 (0.002430)	0.038928 (0.002802)	0.180438 (0.058308)	8.343e-10
Biological yield	0.007063 (0.001939)	0.026135 (0.001962)	0.270261 (0.064620)	4.602e-30
Flower colour	0.007769 (0.001459)	0.014672 (0.001278)	0.529494 (0.064950)	3.048e-68
Peduncle colour	0.137912 (0.018070)	0.158668 (0.016023)	0.869184 (0.033109)	2.322e-95
Days from beginning of germination to end of germination	0.000926 (0.000393)	0.010681 (0.000753)	0.086721 (0.036112)	5.78e-10
Days from beginning of germination to beginning of flowering	0.001010 (0.000306)	0.005561 (0.000399)	0.181642 (0.051013)	1.856e-22

Days from beginning of germination to beginning of maturation	0.000726 (0.000364)	0.009810 (0.000691)	0.073985 (0.036717)	1.148e-06
Days from beginning of germination to end of flowering	0.001100 (0.000508)	0.011691 (0.000824)	0.094063 (0.042638)	9.032e-06
Days from beginning of germination to end of maturation	0.001951 (0.000732)	0.015608 (0.001105)	0.124988 (0.045186)	7.725e-13
Height of lower pod attachment	0.002566 (0.000949)	0.019848 (0.001405)	0.129292 (0.045960)	3.29e-13
Pod length	0.002771 (0.000623)	0.007645 (0.000600)	0.362526 (0.064794)	8.567e-59
Pod width	0.009149 (0.001987)	0.022513 (0.001824)	0.406368 (0.067230)	8.524e-63
Pod drop	0.008981 (0.006800)	0.243616 (0.017166)	0.036866 (0.027985)	0.01196
Pod shape	0.006080 (0.001589)	0.023356 (0.001732)	0.260306 (0.059463)	6.084e-27
Pod shattering	0.010091	0.242713	0.041576	0.00761

	(0.006941)	(0.017089)	(0.028629)	
Number of pods per plant	0.001360 (0.000720)	0.019881 (0.001399)	0.068411 (0.035921)	0.0004563
Plant height	0.004750 (0.001152)	0.013878 (0.001086)	0.342281 (0.067707)	3.511e-46
Seed colour	0.074101 (0.010658)	0.091479 (0.009254)	0.810035 (0.042686)	6.139e-82
Leaf size	0.021757 (0.005475)	0.063592 (0.005010)	0.342136 (0.070480)	2.716e-37
Number of seeds per plant	0.001857 (0.000869)	0.024829 (0.001747)	0.074797 (0.034589)	3.575e-08
Seed shape	0.011722 (0.003608)	0.052466 (0.003843)	0.223428 (0.062258)	3.636e-14
The nature of the branching (secondary branches)	0.000916 (0.000500)	0.021587 (0.001519)	0.042412 (0.023111)	6.28e-06
The nature of the branching (primary branches)	0.023745 (0.009515)	0.206547 (0.014567)	0.114962 (0.044669)	3.999e-10
Stem branchness	0.001297 (0.001255)	0.071521 (0.005049)	0.018130 (0.017624)	0.04729
Stem colour	0.069536	0.111903	0.621397	2.247e-75

	(0.012038)	(0.010346)	(0.060977)	
Length of the primary branches	0.005564 (0.002460)	0.077457 (0.005443)	0.071828 (0.031351)	4.17e-11
Seeds weight per plant	0.002587 (0.001057)	0.023979 (0.001691)	0.107894 (0.042904)	6.778e-10
Thousand seeds weight	0.015501 (0.002718)	0.024210 (0.002301)	0.640276 (0.061915)	7.008e-91
Plant weight without pods	0.006433 (0.001432)	0.016548 (0.001326)	0.388721 (0.067201)	1.047e-42

* - Standard Error.

Table S7 Significant SNPs for phenotypic traits and bioclimatic variables. GWAS analysis with the first 8 PCA axes scores used as covariates and GWAS analysis including the first 8 PCA axes scores and the first 2 PCoA axes scores as covariates for all traits. Annotation of significant associated markers was performed using SNPEff program, Legume information system (LIS) and LegumeIP databases.

(see TableS7.xlsx)

Table S8 Haploblocks inferred by Haploview tools.

(see TableS8.xlsx)

Table S9 List of haploblocks enriched for SNPs associated with phenotypes and bioclimatic variables.

Haploblocks*	Variable
Ca6_Block_9, Ca2_Block_21, Ca4_Block_69, Ca8_Block_9	AscoRes
Ca1_Block_38, Ca4_Block_9, Ca4_Block_13, Ca4_Block_14, Ca5_Block_4, Ca3_Block_2, Ca7_Block_12, Ca3_Block_7, Ca1_Block_19	BegFloEndFlo
Ca4_Block_8, Ca4_Block_74, Ca7_Block_12, Ca1_Block_27, Ca8_Block_3	BegMatuEndMatu
Ca1_Block_25, Ca8_Block_6, Ca4_Block_8	BushShape
Ca4_Block_8, Ca8_Block_6, Ca4_Block_5, Ca1_Block_28	Byld
Ca1_Block_38, Ca3_Block_2, Ca4_Block_9, Ca4_Block_13, Ca4_Block_14, Ca4_Block_15, Ca5_Block_4, Ca7_Block_12, Ca5_Block_7, Ca3_Block_7, Ca4_Block_12, Ca4_Block_69	EndFloBegMatu
Ca4_Block_5, Ca8_Block_7, Ca1_Block_2, Ca1_Block_25, Ca4_Block_23, Ca6_Block_9, Ca4_Block_1, Ca4_Block_4, Ca4_Block_12, Ca4_Block_67	FloCol
Ca6_Block_16, Ca8_Block_7, Ca4_Block_30, Ca7_Block_1, Ca6_Block_17	FlowstemColo
Ca4_Block_9, Ca4_Block_15, Ca8_Block_6, Ca4_Block_23, Ca4_Block_67, Ca4_Block_68, Ca1_Block_27, Ca6_Block_3, Ca8_Block_3, Ca5_Block_4, Ca1_Block_19	Germ
Ca3_Block_16, Ca4_Block_8, Ca6_Block_3, Ca6_Block_9, Ca8_Block_6, Ca1_Block_28, Ca4_Block_9, Ca4_Block_13, Ca1_Block_24, Ca6_Block_4, Ca8_Block_9, Ca6_Block_10,	GermBegFlo

Ca3_Block_7, Ca8_Block_3	
Ca1_Block_25, Ca2_Block_21, Ca5_Block_4, Ca1_Block_27, Ca8_Block_3, Ca4_Block_74, Ca6_Block_4, Ca4_Block_4	Hlp
Ca1_Block_25, Ca7_Block_4, Ca5_Block_4, Ca2_Block_8	PDL
Ca4_Block_67, Ca5_Block_4, Ca7_Block_4, Ca8_Block_9, Ca4_Block_68, Ca7_Block_5, Ca1_Block_18, Ca3_Block_4, Ca4_Block_66	PDW
Ca1_Block_28, Ca4_Block_5, Ca5_Block_4, Ca3_Block_4, Ca4_Block_12, Ca6_Block_9, Ca4_Block_8, Ca7_Block_11, Ca8_Block_9	PPP
Ca3_Block_2, Ca3_Block_11, Ca8_Block_6, Ca7_Block_1, Ca6_Block_2, Ca6_Block_29	PodDrop
Ca3_Block_2, Ca4_Block_8, Ca4_Block_54, Ca4_Block_7, Ca7_Block_1, Ca6_Block_4, Ca1_Block_18, Ca8_Block_3, Ca4_Block_23	PodSH
Ca3_Block_2, Ca4_Block_9, Ca4_Block_4, Ca4_Block_71, Ca6_Block_2, Ca6_Block_4, Ca4_Block_8, Ca2_Block_8	PodShat
Ca4_Block_5, Ca4_Block_54, Ca2_Block_21, Ca5_Block_4, Ca8_Block_6, Ca1_Block_28, Ca4_Block_8, Ca4_Block_13	PodsWeight
Ca2_Block_21, Ca4_Block_1, Ca4_Block_8, Ca8_Block_6, Ca3_Block_16, Ca1_Block_25, Ca1_Block_28, Ca4_Block_66, Ca4_Block_7, Ca4_Block_23	Ptht
Ca2_Block_8, Ca8_Block_7, Ca4_Block_4, Ca2_Block_21,	SCO

Ca4_Block_67	
Ca1_Block_28, Ca3_Block_4, Ca4_Block_5, Ca4_Block_8, Ca5_Block_4, Ca7_Block_12, Ca4_Block_40, Ca4_Block_67	SPP
Ca4_Block_7, Ca4_Block_23, Ca5_Block_7, Ca1_Block_21, Ca4_Block_71, Ca4_Block_4	SSH
Ca4_Block_5, Ca5_Block_4, Ca4_Block_7, Ca3_Block_4, Ca4_Block_54, Ca4_Block_8, Ca3_Block_7, Ca3_Block_17, Ca1_Block_28, Ca8_Block_6	SYDS
Ca1_Block_27, Ca4_Block_13, Ca6_Block_10, Ca3_Block_4, Ca6_Block_17, Ca1_Block_18, Ca1_Block_25, Ca5_Block_4, Ca4_Block_29, Ca4_Block_4, Ca4_Block_67, Ca4_Block_8, Ca6_Block_9	SizeLeaf
Ca2_Block_21, Ca4_Block_12	StemBranch2order
Ca3_Block_4, Ca5_Block_7, Ca8_Block_7	StemBranchinnes1ord
Ca8_Block_7, Ca4_Block_1, Ca7_Block_12, Ca1_Block_37, Ca8_Block_9	StemBranchness
Ca8_Block_7, Ca8_Block_9, Ca3_Block_11, Ca6_Block_17, Ca1_Block_27, Ca4_Block_5, Ca4_Block_13, Ca6_Block_16, Ca8_Block_3	StemColo
Ca1_Block_25, Ca4_Block_18, Ca1_Block_5	StemLength1order
Ca1_Block_25, Ca1_Block_28, Ca8_Block_6, Ca1_Block_27, Ca4_Block_18, Ca8_Block_9, Ca1_Block_18, Ca4_Block_23, Ca4_Block_67, Ca7_Block_1, Ca1_Block_37, Ca4_Block_7,	TSW

Ca1_Block_40	
Ca4_Block_8, Ca8_Block_6, Ca3_Block_16, Ca4_Block_23, Ca4_Block_4	WpWp
Ca6_Block_28, Ca8_Block_6, Ca6_Block_2, Ca3_Block_2	BIO_1
Ca6_Block_3, Ca4_Block_7, Ca6_Block_2, Ca8_Block_9, Ca1_Block_25	BIO_2
Ca8_Block_3, Ca8_Block_9, Ca3_Block_16, Ca7_Block_12	BIO_3
Ca4_Block_4, Ca4_Block_9	BIO_4
Ca3_Block_2, Ca8_Block_6, Ca1_Block_21, Ca3_Block_16, Ca3_Block_7, Ca6_Block_9	BIO_5
Ca4_Block_9, Ca6_Block_2, Ca4_Block_4, Ca1_Block_27	BIO_6
Ca4_Block_9, Ca6_Block_2, Ca4_Block_4, Ca7_Block_4, Ca4_Block_11	BIO_7
Ca6_Block_2, Ca6_Block_28, Ca5_Block_4	BIO_8
Ca3_Block_2, Ca8_Block_6, Ca1_Block_21	BIO_{10}
Ca6_Block_2, Ca4_Block_4, Ca8_Block_6, Ca5_Block_4	BIO_{11}
Ca6_Block_28, Ca6_Block_2, Ca4_Block_23, Ca4_Block_18, Ca4_Block_9, Ca5_Block_4	BIO_{12}
Ca5_Block_4, Ca6_Block_2, Ca6_Block_28, Ca3_Block_7	BIO_{13}
Ca1_Block_25, Ca4_Block_1, Ca4_Block_7, Ca4_Block_8, Ca8_Block_6, Ca6_Block_3, Ca6_Block_2, Ca1_Block_21, Ca4_Block_66	BIO_{14}

Ca1_Block_28, Ca1_Block_21, Ca5_Block_4	BIO_{15}
Ca5_Block_4, Ca6_Block_2, Ca6_Block_28, Ca4_Block_23, Ca3_Block_7	BIO_{16}
Ca4_Block_7, Ca4_Block_8, Ca6_Block_2, Ca6_Block_3, Ca8_Block_6, Ca3_Block_17, Ca4_Block_66, Ca1_Block_25, Ca4_Block_1, Ca1_Block_21	BIO_{17}
Ca4_Block_23, Ca6_Block_2, Ca6_Block_28, Ca4_Block_74	BIO_{18}
Ca2_Block_21, Ca5_Block_7, Ca6_Block_3, Ca7_Block_1, Ca1_Block_18, Ca6_Block_9	BIO_{19}
Ca6_Block_28, Ca6_Block_2, Ca4_Block_15, Ca4_Block_67, Ca8_Block_6, Ca6_Block_3	DEM

* - Haploblocks' coordinates are in Table S8.

Table S10 Groups of phenotypes and variables similar in sets of enriched haploblocks.

**Group**	**Variables**
Group1	EndFloBegMatu, BegFloEndFlo
Group2	BIO_1, BIO_4, BIO_6, BIO_7, BIO_8, BIO_{11}, BIO_{12}, BIO_{13}, BIO_{16}, BIO_{18}, DEM, PodShat, PodDrop
SubGroup3	FlowstemColo, StemColo, SCO, FloCol, StemBranchinnes1ord, StemBranchness
SubGroup4	BIO_{19}, AscoRes, StemBranch2order
Group5	BIO_2, BIO_5, BIO_{10}, BIO_{14}, BIO_{17}, WpWp, Ptht, BushShape
Group6	Byld, PodsWeigth, SYDS, SPP, PPP

Group7	GermBegFlo, PodSH, TSW, Germ, Hlp, SizeLeaf, PDW, PDL, BIO_{15}

Table S11 SNPs detected in current study and QTLs and SNPs from previous studies.

(see TableS11.xlsx)

Table S12 Haploblocks enriched for SNPs associated with phenotypes and bioclimatic variables detected in current study and QTLs and SNPs from previous studies.

(see TableS12.xlsx)
