# Supplementary material for: Genomic Analysis of Vavilov’s Historic Chickpea Landraces Reveals Footprints of Environmental and Human Selection
Source: Int J Mol Sci. 2020 May 31;21(11):3952. doi: 10.3390/ijms21113952 (PMC7313079; doi:10.3390/ijms21113952)
Supplement: Supplementary file 1 [file ijms-21-03952-s001.zip › Supplementary Texts S1-S3.pdf]

Supplementary Text S1 Phenotyping

Phenotyping of the 407 chickpea accessions was conducted at the VIR Kuban experimental station from April 1 to November 30, 2016. The Kuban station is situated at the steppe zone of Nearkuban flatlands, 80 km away from the Kavkaz foothills. The typical soils in these regions are nearkavkaz blacklands, with fertile soil depths of 140-150 cm and slightly alkaline pH. The humus horizons depth is typically between 130-170 cm and humus content of 3.6-4.6%. The climate of the station is characterized by suboptimal rainfall, and high fluctuations of all the climatic parameters. The climate is characterized as moderate-continental, with hot summers. Median temperature of the coldest month (January) is -2.6 °C, and of the hottest month (July) 23 °C. Total yearly rainfall is 565 mm. These climatic conditions are well suited for chickpea cultivation.

In the autumn of 2015, after harvesting a preceding crop of winter wheat, a continuous disking of the soil was carried out. In October the soil was plowed to a depth of 25 cm. In the spring of 2016 three-fold cultivation of the field was carried out. Prior to sowing, on April 22 the section for chickpeas was treated with "Stompe" herbicide after laying out the planting design, followed by embedding the herbicide by continuous cultivation. Sowing of all 407 landraces of chickpea was conducted on April 28 in one day with the help of manual seeders according to the scheme of 400 × 5 × 60 cm, with the depth of seeding of 5 cm. The onset of germination was observed on May 6, 2016 and full shoots were observed on most specimens on May 9. During chickpea cultivation, the plants were hand treated four times to remove all undesirable weeds.

During the vegetative period, thirty six phenological, morphological, agronomical, and biological descriptors were measured. At the time of the harvesting, the full architectural analyses had been executed for 5 randomly chosen plants per genotype. The following phenotypes were recorded in the field: date of beginning seedlings (10% plants emerged), date of full of seedlings (75% plants), date of beginning of flowering (10% plants), date of full of flowering (75% plants), date of end of flowering, date of beginning of ripening, date of full

maturation, flower color, stem color, bush shape, leaflet size, peduncle color, degree of *Ascochyta* blight damage, degree of *Ascochyta* blight resistance, stem branching, length of the primary stem branches, number of the primary stem branches, number of the secondary stem branches, pod shedding, and pod dehiscence. From dates of starting and ending of germination, flowering and maturation we obtained the number of days for each period. The architectural analysis was executed for plant height, height of lowest pod attachment, biological yield, weight of plant without pods, pods weight, pods per plant, seeds per plant, weight of seeds per plant, pod shape, pod length, pod width, seed shape, seed color, and one thousand seeds weight. Phenotype abbreviations and units of measurement are in Supplementary Table S4.

Supplementary Text S2 Details of GBS and SNP calling

The procedure from von Wettberg et al. (2018) was followed. Briefly, genomic DNA was digested with two restriction enzymes, *Hind*III and *Nla*III. A "barcode" adapter was ligated to the *Hind*III end, allowing pooling the samples. Size selected fragments were amplified through 14 rounds of PCR and products were sequenced as 100 base reads on an Illumina HiSeq4000 at the University of California at Davis Genome Core. All Illumina data is available from the National Center for Biotechnology database under the BioProject PRJNA388691. Illumina reads were mapped to the *Cicer arietinum* CDCFrontier v1.0 (Varshney et al. 2013) using BWA MEM (Li and Durbin 2010) under default mapping parameters. Polymorphisms were called using the GATK pipeline (McKenna et al. 2010), which considers indel realignment and base quality score recalibration, and calls variants across all samples simultaneously through the HaplotypeCaller program in GATK. Variants were filtered using standard hard filtering parameters according to GATK Best Practices recommendation (DePristo et al. 2011; Van der Auwera et al. 2013). SNP calls were retained with Mapping Quality (MQ) > 37 and Quality by Depth (QD) > 24. Both metrics take into consideration the quality of the mapping and genotype calls to ensure that only those with highest confidence were used. SNPs were also filtered to retain those with

MQRankSum < |2.0|, which ensures that there is no difference in the Mapping Quality scores between alleles. This filtering removed nearly 60% of variant sites reported by GATK and only retained those that pass all three criteria, resulting in 56,855 SNPs. SNPs were further filtered using VCFtools (Danecek et al. 2011) to require minor allele frequency (MAF) >3% and genotype call-rate >90%. 2,579 SNPs in 407 accessions passed all filters remained for further analysis.

Supplementary Text S3 Bioclimatic Analysis

Environmental data were downloaded in the form of GIS layers from the WorldClim – Global Climate Data (Hijmans et al. 2005a) and USGS (USGS.gov). Layers describe a combination of current conditions and interpolations of observed values that span 1950–2005. Digital Elevation Data, GTOPO30, were downloaded from the NASA – USGS LP DAAC archive (Global 30 Arc-Second Elevation) (Barto 2000). The ‘land suitability for cultivation’ dataset was downloaded from the Nelson Institute, Centre for Sustainability and the Global Environment, University of Wisconsin-Madison, (SAGE) (Center for Sustainability and the Global Environment; Ramankutty et al. 2002). Koppen-Geiger climate zones were acquired from the Center for International Development at Harvard University (Geography Data Sets). Data layers that came in vector format were rasterized to match a spatial resolution of 30 sec, which corresponds to approximately 1 sq km at the equator. The data were interpolated from average monthly recordings from weather stations (Hijmans et al. 2005b). Both environmental layers and accession points were in the Longitude/Latitude coordinate system with WGS datum. The environmental values for each accession point were extracted from corresponding layers using the extraction tool in ESRI ArcGIS software (Esri: GIS Mapping Software). Overall, nineteen quantitative bioclimatic variables were used in analysis, their abbreviations and units of measurement are in Supplementary Table S2.

References

1. Barto KP (2000) Model GTOPO30 Data in ArcView GIS. Esri 48–51.

2. DePristo MA, Banks E, Poplin R, Garimella KV, Maguire JR, Hartl C et al. (2011) A framework for variation discovery and genotyping using next-generation DNA sequencing data. Nat. Genet. 43:491–498.

3. Center for Sustainability and the Global Environment | SAGE | University of Wisconsin-Madison. [WWW document] URL http://nelson.wisc.edu/sage/data-and-models/atlas/index.php. [Accessed: 16th May 2017].

4. Esri: GIS Mapping Software, Spatial Data Analytics & Location Platform. [WWW document] URL http://www.esri.com/. [Accessed: 16th May 2017].

5. Hijmans RJ, Cameron SE, Parra JL, Jones PG, Jarvis A (2005) The WorldClim interpolated global terrestrial climate surfaces. http://www.worldclim.org, doi:10.1002/joc.1276.

6. Hijmans RJ, Cameron SE, Parra JL, Jones PG, Jarvis A (2005) Very high resolution interpolated climate surfaces for global land areas. Int. J. Climatol. 25:1965–1978.

7. Li H, Durbin R (2010) Fast and accurate long-read alignment with Burrows-Wheeler transform. Bioinformatics 26:589–595.

8. Ramankutty N, Foley JA, Norman J, McSweeney K (2002) The global distribution of cultivable lands: Current patterns and sensitivity to possible climate change. Glob. Ecol. Biogeogr. 11:377–392.

9. USGS.gov | Science for a changing world. [WWW document] URL https://www.usgs.gov/. [Accessed: 16th May 2017].

10. Van der Auwera GA, Carneiro MO, Hartl C, Poplin R, Del Angel G, Levy-Moonshine A et al. (2013) From fastQ data to high-confidence variant calls: The genome analysis

toolkit best practices pipeline. Curr. Protoc. Bioinforma. 43:11.10.1-33.

11. Varshney RK, Song C, Saxena RK, Azam S, Yu S, Sharpe AG et al. (2013) Draft genome sequence of chickpea (*Cicer arietinum*) provides a resource for trait improvement. Nat. Biotechnol. 31:240–246.
